# Enhancing Executive Function in Adolescents With ADHD: Preliminary Findings From a Multicomponent Intervention

**DOI:** 10.1155/oti/6621288

**Published:** 2026-06-10

**Authors:** Elisavet Pavlou Papagianni, Eleni Theodorou

**Affiliations:** ^1^ Department of Rehabilitation Sciences, Cyprus University of Technology, Limassol, Cyprus, cut.ac.cy

## Abstract

**Background:**

Attention deficit hyperactivity disorder (ADHD) in adolescence is associated with executive function difficulties, which compromise academic performance, daily functioning, and adaptive behaviors. Occupational therapy is aimed at improving EF and occupation and participation impairments. This study presents preliminary findings on the effectiveness of a multicomponent intervention protocol designed to enhance cognitive abilities in adolescents with ADHD.

**Methods:**

A quasi‐experimental design with one‐group, pretest–posttest, was used to evaluate the impact of the MOTI, which included computer‐based cognitive training, academic skills training, and complementary but not mandatory a parents′ psychoeducation group. Fifty‐four adolescents aged 13–18 participated. Preintervention and postintervention assessments measured cognitive abilities and mental health indicators.

**Results:**

Significant improvements were observed in working memory (digit span, CAB), mental flexibility (Trail Making B), and processing speed (Trail Making A). Omissions on the continuous attention test decreased (*p* = 0.046), and students self‐reported better planning and organizing skills (*p* = 0.034).

**Conclusions:**

Evidence supports the effectiveness of the multicomponent intervention protocol (MOTI) in improving executive functions in adolescents aged 12–18 with ADHD. Initial analyses of the cognitive and academic skills training component reveal positive outcomes in targeted executive functions, reinforcing this multicomponent intervention as a valuable occupational therapy approach for this population.

## 1. Introduction

Attention deficit hyperactivity disorder (ADHD) is a neurodevelopmental disorder characterized by persistent patterns of inattention, hyperactivity, impulsivity, and disorganization [1]. Emerging typically in childhood and often persisting into adulthood [2], ADHD has been closely associated with deficits in executive functioning (EF)—the suite of cognitive processes essential for goal‐oriented behavior, including attention control, planning, memory, and decision‐making [3–5].

Individuals with ADHD frequently struggle with adaptive functioning and developmental transitions, impacting their performance in day‐to‐day tasks [6, 7]. These challenges are particularly pronounced during adolescence—a critical period marked by social, academic, and emotional transformation. Without proper support, adolescents with ADHD are at heightened risk for academic underachievement, social difficulties, injuries, and substance use, contributing to a reduced quality of life [2, 8].

Executive function difficulties are not universal within the population of individuals with ADHD in the sense that not all individuals present with difficulties in EF, and among those who do, there is great heterogeneity in the presentation of EF status [9]. Yet, deficits in EF are deemed to be responsible for the manifestation of the ADHD phenotype and to contribute to difficulties young people with ADHD face with academic and social/emotional functioning [9, 10].

The most often reported difficulties with EF observed in adolescents with ADHD are in working memory (WM), inhibition control and planning/organization [9]. Specifically, inhibitory control (IC) is consistently highlighted as most impaired in individuals with ADHD [11, 12], triggering cascading effects on WM, emotional regulation, and adaptive behavior. The term inhibition (or IC) describes “the ability to stop a prepotent response in favor of a more adaptive response” [13]. Similarly, planning—defined as the ability to design strategic sequences of actions toward achieving goals—remains a vital yet often compromised function in ADHD populations, with broad academic and psychosocial implications [14–16].

Deficits in EF, especially in areas such as attention, planning, and memory, manifest in difficulties with both activities of daily living (ADLs) and instrumental activities of daily living (IADLs), ranging from basic self‐care to complex tasks like household management and financial planning [17]. These impairments not only hinder school performance but also reduce participation in leisure, social, and home settings [8, 18].

When intact, EF components integrate to facilitate complex performance and successful engagement in novel and dynamic occupations, with a constant adaptation of the behavior according to the changing environments [19, 20]. However, in situations where EF deficiencies are present as in neurodevelopmental disorders, difficulties in several areas such as cognition, social, and academic development are present and individuals struggle to complete daily life tasks, as they present as disorganized, forgetful, unable to multitask, and to self‐regulate their behavior [16, 20].

As Molitor et al. [9] note, maturation of executive function in adolescence follows the increase of academic and social demands on these skills, in the daily lives of adolescents—especially as they transit to middle school [21]. Focusing on the role of the student or the occupation of learning, we know that in order for these to be successful, a student should implement self‐regulated learning strategies which include but are not limited to organization and time management, active schoolwork engagement, and cognitive and intellectual skills (WM, problem‐solving skills, etc.) [22–24]. In order for a student to “successfully” participate in school life and learning, they are required to perform complex cognitive activities, exhibit goal‐directed behaviors, concentrate, attend to the teacher, understand and follow rules, and control their impulses [25]. Further, they need to remember details to complete assignments, plan the completion of the assignments, and follow the instructions in order to complete and handle them [26].

IC, which is the ability to suppress automatic and/or dominant responses and avoid turning the focus on distractions and irrelevant stimuli [27, 28] is crucial in the daily performance of occupations like attending school classes, being able to concentrate on the teacher’s lecture, and resisting distractions [27] like those coming from classmates. During class, WM is crucial in enabling the student to retain relevant and ignore irrelevant information to form answers to a teacher′s question or to take accurate notes. Cognitive flexibility is also an important element as students are frequently asked to switch between different tasks [29]—switch their behavior at the end of the break to go back to class, listen to the teacher, and copy from the board, and follow a different set of rules to solve problems in mathematics, physics, or other subjects, and so on.

Despite years of research, consensus around the most effective treatment strategies for ADHD and associated EF deficits remains elusive [12]. Although short‐term benefits are observed across a range of pharmacological and behavioral treatments, long‐term efficacy remains uncertain [29–31].

Nevertheless, evidence suggests that individuals receiving any form of intervention—cognitive, behavioral, or pharmacological—tend to exhibit better long‐term outcomes than untreated peers [32]. Cognitive training, defined as the intentional practice of cognitive functions [29], is frequently cited as a cornerstone in managing ADHD‐related difficulties.

Occupational therapy (OT) interventions, specifically tailored to adolescents with ADHD, offer promise in enhancing participation across meaningful activities, including self‐care, education, leisure, and social engagement—thereby supporting overall well‐being [8, 33].

Emerging methods, within the field of OT, such as central executive training (CET) and IC training, have gained recognition for their targeted impact on impulse control, WM, and academic outcomes in children aged 8–13 [34–36]. These techniques demonstrate both near and far‐transfer benefits, indicating broader applicability to real‐life contexts.

Another method that has been recognized for its effectiveness is cognitive‐functional (Cog‐Fun) OT intervention, which is described as an occupation‐based approach designed to improve cognitive functioning in the context of meaningful daily activities [7, 37]. Cog‐Fun has been studied for its effectiveness in adolescents aged 12–17, yielding positive effects on occupational performance and EF [7].

This study examines the efficacy of a multicomponent occupational therapy intervention protocol (MOTI) designed specifically for adolescents aged 13–18 diagnosed with ADHD. Drawing on evidence‐based guidelines and established best practices, the MOTI combines individualized cognitive training with ecologically relevant skill‐building modules, focusing directly on academic difficulties and indirectly on everyday functional challenges.

Design of the intervention under investigation was based on some key features derived from literature, as follows:

• Individualized training load: Adaptive software delivers short, customizable training sessions each), promoting engagement and sustained effort [30, 31].
• Ecological validity: Integration of real‐life academic planning and study strategies enhances far‐transfer outcomes [38, 39].
• Developmental appropriateness: Focus on adolescence—a pivotal phase for future autonomy and academic adjustment [31, 32].
• Comprehensive targeting: Simultaneous focus on multiple EF domains (WM, inhibition, planning, and flexibility) reflects the integrated nature of everyday demands [40].

To guide our study, the following hypotheses were formed:

**Hypothesis 1.**

There will be a significant improvement in working memory scores because of the MIP, as measured preintervention and postintervention.

**Hypothesis 2.**

There will be a significant improvement in mental flexibility scores as a result of the MIP as measured preintervention and postintervention.

**Hypothesis 3.**

There will be a significant improvement in inhibitory control scores as a result of the MIP as measured preintervention and postintervention.

**Hypothesis 4.**

There will be a significant improvement in general cognitive abilities measured as processing speed and reaction time, as a result of the MIP and as measured preintervention and postintervention.

## 2. Methods

### 2.1. Design and Procedures

The research study was approved by the Cyprus National Bioethics Committee—reg. Number: CNBC/EP/2021/10 (11/06/2021) and the Centre for Academic Research and Evaluation of the Cyprus Ministry of Education, Sports and Youth—reg. Number: 7.15.01.25.8.2/6 (11/08/2021). Signed informed consent for participation was obtained from both the adolescents and their parents or legal guardians. The study followed a six‐step procedure as described in Table 1 below.

**Table 1:** Study process chart.

Step 1 An open call for participation was published in social media, send to parents associations of every public and private secondary school in Cyprus and to professional associations (Cyprus Association of Occupational Therapists, Association of Speech‐Language Pathologists‐Speech Therapists in Cyprus, etc.) with a plead to inform their members and to ADHD‐Cyprus (a nongovernmental organization for ADHD). Parents expressed an initial interest for participation; they were provided with information and procedures and an oral consent for future communication was obtained.
Step 2 Screening by parents′ questionnaires and scales ADHD Greek Scale [41, 42]. Achenbach System Of Empirically Based Assessment (ASEBA) [43–45]. Parents interview—a questionnaire gathering demographic and developmental progress data, current strengths, and difficulties. Designed for the purposes of the study.
Step 3 Assessment meeting Adolescent assessment was done by the researcher OT. Student′s interview—a questionnaire gathering data about the “lived experience” of the students, their strengths and difficulties, personal goals, academic and social participation, and so on. Students and their parents were provided with details for the study, given time to ask questions, informed about their rights and given instructions for the next steps. A written informed consent was signed by both parents as the law provides, and the student.
Step 4 Preintervention measures Adolescent assessment was done by the researcher OT. ASEBA [43, 44]. Rosenberg Self‐Esteem Scale [46, 47]. Trail Making Test Part A and B [48, 49]. Continuous attention test [50]. Digit span test (forward and backward) [51, 52]. Cognitive Assessment Battery by Cognifit [53].
Step 5 Intervention: For adolescent students: 1.1 Cognitive training: Online using Cognifit, from their settings and at their own pace. Instructed to take four sessions X 25 min per week. 1.2 Academic skills training: Online meetings with the researcher OT, started after the completion of 3 weeks of cognitive training. Five sessions X 45 min. For parents: Parents′ psychoeducation group. Options for in vivo and online sessions. Five group sessions are held weekly X 90 min, conducted by the researcher OT. Parents were allocated to the groups based on their convenience, after their child′s intervention was completed (or was at the final week).
Step 6 Postintervention measures: Completed no more than 3 weeks after the completion of the intervention. Parents′ questionnaires and scales: ADHD Greek Scale [41]. ASEBA [44]. Students′ questionnaires and scales. ASEBA [44]. Rosenberg Self‐Esteem Scale [46, 47]. Trail Making Test Part A and B [49, 54]. Continuous attention test [50]. Digit span test (forward and backwards) [51, 52]. Cognitive Assessment Battery by Cognifit [55]. Students′ interview: A questionnaire designed for the purpose of the current study, documenting strengths and difficulties after the intervention in accordance to those recorded preintervention.

The study followed a quasi‐experimental design, with a one‐group pretest–posttest design with the aim of evaluating the effectiveness of a novel intervention protocol that combines cognitive training, skills training, and parents′ psychoeducation. The chosen design permits focus on the effectiveness of the proposed intervention rather than comparison with other interventions or with nontreatment, which was out of the aims of the research project. Providing novel interventions, which are evidence‐based and can serve as alternative choices that best suit individual needs was valued of utmost importance rather than comparing interventions, at least at the current stage.

One qualified occupational therapist (the researcher) delivered the intervention protocol under the supervision of the Department of Rehabilitation Sciences of the Cyprus University of Technology. Sessions were hybrid in the sense that a part of them was delivered in person and a part was delivered online.

### 2.2. Participants

Participants were adolescents aged 13–18 years with a formal diagnosis of ADHD. Recruitment was conducted on a voluntary basis through a public call for participation disseminated via social media, nongovernmental organizations willing to share the information with their members or post it on their platforms, and through parent associations affiliated with public and private secondary and higher schools across Cyprus.

Inclusion criteria were as follows:

a. Adolescents must have a confirmed diagnosis of ADHD, verified either by a child psychiatrist or by the Educational Psychology Service of the Cyprus Ministry of Education, Sports and Youth.
b. They must be between 13 and 18 years of age and enrolled in mainstream educational settings.
c. They must demonstrate fluency in both verbal and written communication in either Greek or English.

Exclusion criteria included the following:

a. A diagnosis of intellectual disability or severe learning difficulties that significantly impair cognitive functioning.
b. Enrollment in special education programs.
c. Current use of medication known to affect cognitive performance at the time of the study.
d. For participants undergoing pharmacological treatment, an unstable medication regimen within the 3 months preceding enrollment.

A total of 54 adolescent students and their parents participated in the study. The sample comprised 36 males (66.8%) and 18 females (33.3%), with a mean age of 14.3 years. The majority of participants (72.7%, *N* = 40) had not previously received any form of treatment for ADHD. Among those who had, 12.7% (*N* = 7) were undergoing pharmacological treatment, whereas 10.8% (*N* = 6) were receiving psychosocial interventions and/or educational support.

### 2.3. Intervention

The primary objective of the MOTI program was to strengthen cognitive functioning through an intervention designed to be seamlessly incorporated into daily routines, thereby promoting increased engagement in meaningful activities among adolescent students.

Each participant received individualized intervention administered by the researcher, a qualified occupational therapist with extensive clinical experience in the field. The intervention followed a hybrid format: skills training was delivered one‐on‐one, either in person or online, whereas cognitive training was conducted via a specialized computer‐based platform, which is described in detail in subsequent sections.

MOTI is tailored for adolescents aged 13–18, a developmental window that aligns with key educational transitions—from primary to secondary school around age 13, and from secondary education to higher education or entry into the workforce around age 18. The program comprises three core components: (a) cognitive training, (b) skills training, and (c) parent psychoeducation. These elements are informed by evidence‐based practices and insights from prior research on ADHD interventions, collectively offering a comprehensive and holistic approach to supporting adolescent students.

#### 2.3.1. Description of the MOTI Components

##### 2.3.1.1. Cognitive Training

Cognitive training represents the most extensive component of the MOTI program. Delivered through a computer‐based format, it offers flexibility by allowing participants to engage in sessions at their convenience. The training was conducted via the Cognifit Platform, enabling adolescents to log in at any time and complete 25‐min sessions composed of activities selected automatically.

These sessions are highly individualized, with both the type of activities and their difficulty level dynamically adjusted based on each participant′s initial assessment and ongoing performance—closely mirroring the adaptive nature of real‐life OT. The platform is user‐friendly and compatible with various devices, including tablets, laptops, and desktop computers. It provides instructions in Greek (among other languages), and most executive function training activities are either nonverbal or appropriately translated into both Greek and English.

Cognifit is chosen by researchers for research studies, including studies with adolescents and people with ADHD or other neurodevelopmental disorders as well as people with EF difficulties. Studies including children and adolescents with ADHD concluded that Cognifit cognitive training had positive effects on executive function with far‐transfer effect, for example, on reading abilities and academic performance (e.g., [56, 57].

##### 2.3.1.2. Academic Skills Training

For the purposes of the MOTI and the current project, the term “academic skills” is used to describe cognitive skills and techniques that are considered to be critical in academic performance. The term is not used to refer to mathematical or reading skills.

The second component is perfectly described by [55], who mention that “skills on learning‐how‐to‐learn enable individuals to actively engage in and sustain their academic life. It involves effectively organizing one′s learning process, managing time and information efficiently. Through this process, students learn to set goals, identify key information, recognize patterns, prioritize tasks, and seek assistance when needed” [55]. Ultimately, these competencies empower students to become self‐directed learners who take ownership of their academic participation and performance.

The academic skills training component, consisting of six sessions of 45 min in duration carried out once or twice per week—based on each student′s preference, either in person or on‐line (again based on each student′s preference). The specific goals of the AST included planning, organizing, and prioritizing. Students were encouraged to set their personal goals in regard to their performance (e.g., what grades would be satisfying for them for each course they had on the semester) and then set their priorities. Then they would be instructed to prepare a weekly planner. The planner should include all their obligations (school and private tutoring, family obligations and/or personal appointments, e.g., visit the dentist) and if any, their prefixed sports/creative/leisure activities (ii) a mark for all their forthcoming tests/exams or deadlines for turning in projects and homework. (iii) Their personal plans (if any) which were desirable but not compulsory, for example, go to a party and (iv) their “free time.” Students were encouraged to choose specific time intervals (during the weekend) which were free of activities or obligations. This unstructured time was considered of great importance for a student′s feeling of relaxation and gave them a sense of mastery of their time.

The next step was to mark for each day of the week (weekdays only) their “study‐time.” This should have a duration of at least 90 min and should be used to complete any homework for school or private tutoring, revision if needed, and “deepening my knowledge” time (please see below for details).

When a student had a forthcoming test or exam or a deadline for homework, they were instructed to select appropriate time and mark it on their weekly planner for revision. A final step was to discuss with each student their concerns regarding their weekly planner and if any concerns were raised, a solution was found.

###### 2.3.1.2.1. Time Management

Along with the “long term” time management, which is trained throughout the preparation of the weekly planner, students were trained in using the Pomodoro Technique to learn how to better manage their time on “short term.”

The Pomodoro technique was first introduced by Francesco Cirillo as a time management technique for students but soon adopted by a great variety of fields as it seems to maximize productivity, minimize distractions and helps avoiding mental fatigue (Pomodoro Technique: 5 Steps for Effective Time Management—Primed To Learn, n.d.) as well as helping with procrastination while increasing motivation [55].

###### 2.3.1.2.2. Sustained Attention and Focus

Students are instructed to set their study environment in a way that minimizes distractions. As a first step, they should recognize their personal major distractions—for some students this is noise, for others it is movements, and so on. They are encouraged to use white‐noise devices or music or even noise‐canceling earbuds. They are also encouraged to ask family members not to distract them for a specific time interval. In addition, they are encouraged to put their desks in a spot that prevents them from looking outside a window or open doors.

###### 2.3.1.2.3. Study Techniques

Students are trained in how to study and take notes, how to prepare flashcards, timelines, relation‐charts, and any other technique that might be useful for each of their academic subjects.

##### 2.3.1.3. Parents′ Psychoeducation

Parents′ psychoeducation served as a measure to increase compliance and commitment of families to intervention. Parents (or guardians/caregivers) were invited to participate in a program consisting of six weekly sessions of a 90‐min duration each, either in person or online, after their children completed the intervention.

Active involvement of parents was deemed to be of crucial importance for the following reasons:

1. A student′s relationship with their peers, parents, and teachers, and the social support one′s environment (including family) provides has a key role in their academic outcomes, performance, and chance of improvement [58].
2. Studies have shown that in families with adolescents with severe symptoms of ADHD there is increased parental stress, marital conflict, higher rates of divorce, and overall poorer family functioning [54, 59].
3. Adolescents with ADHD who are exposed to protective factors such as positive parenting benefit from direct influences that support their overall well‐being [60]. Other protective factors include positive social and family connections, parents′ understanding of growth and developmental changes and need, concrete parental support, and so on. On the other hand, poor parent–child relationships increase the risk of an adolescent with ADHD developing depressive and externalizing symptoms [60].
4. Functional impairments of children with ADHD (especially inattentive type) are related to poor compliance with parental instructions, poor parent–child relationships, and parental reports for using ineffective parenting practices and experiencing higher levels of family stress in comparison to families with children with no ADHD diagnosis [61]. As Haack et al. [53] state, “if parenting influence functional impairment above and beyond the influence of child ADHD symptoms per se, it would provide a rationale for targeting both parenting and child symptoms in treatment” [61].

At this stage in the development of the multicomponent intervention, the focus is primarily on evaluating the effectiveness of cognitive and skills training through a structured, step‐by‐step approach. Consequently, the third component—parents′ psychoeducation—is introduced at a later phase to ensure it does not influence the core outcomes of the intervention. This sequencing reflects the underlying assumption that adolescents should maintain a high degree of autonomy in their therapeutic engagement, while acknowledging the supportive role families can play. Defining parents′ psychoeducation as a mandatory element of the intervention could potentially compromise adolescent autonomy by making their participation contingent upon parental involvement and decision‐making. On the other hand, keeping the parental involvement as complementary and voluntary we expect to strengthen the family′s commitment to the program.

The psychoeducation sessions are interactive and include a power‐point presentation with a selection of relevant topics while providing sufficient time for parents to express their own thoughts and experiences and raise issues and concerns about their personal experience.

Topics of parents′ psychoeducation include milestones of typical development, symptoms and characteristics of ADHD, effective family communication, regulating behavior with the use of appropriate boundaries and communication, treatment options, and support for children and young people with ADHD.

### 2.4. Measures

Measures and assessments of cognitive abilities were taken prior and post intervention. Preintervention measurements were taken one to 2 weeks prior to participation, and postintervention measures were taken upon completion of intervention (the following 5–10 days after completion of intervention).

#### 2.4.1. WM

WM includes (a) the maintenance of relevant to task‐in‐hand information and (b) the dynamic manipulation of the contents of WM, checking for relevance to the task‐in‐hand and replacing nonrelevant information with other more relevant [62, 63]. For the assessment of WM, the following tools were used.

Digit span test is the test requires from the student to repeat a series of digits (numbers) read aloud by the examiner, in its correct order [51, 64]. Three variations were used:

• Forward digit span test: the student should repeat the digits in their correct forward order [51].
• Backwards digit span test: where the students were asked to repeat the digits in a backward order and [51].
• Digit span with distraction: a variation of the digit span test designed by the researcher that is believed to measure mental flexibility and WM. The student, after listening to the digits, needs to answer a simple question like “What are the names of people living with you” and then recall the digits in their correct forward order. The variation is believed to resemble class situations where students are asked to handle information in an environment not free of distractions.

Cognitive assessment battery by Cognifit (https://www.cognifit/) is a brief, comprehensive neuropsychological battery providing the assessment of 22 cognitive skills including executive function components such as WM, inhibition, planning and organizing, and mental flexibility [53]. The battery presents five different tasks (activities) to measure various aspects of memory, including WM. The tests are taken online, and the result is given automatically with an analytical report. It is used preintervention and postintervention, and potential changes in the students′ cognitive profile are evaluated. Better postintervention performance indicates the positive effect of the MOTI.

#### 2.4.2. IC

IC (or inhibition) describes the ability of a person to deliberately inhibit (stop or control) a dominant, automatic or prepotent response that is usually socially undesirable and impulsive in favor of a more acceptable and adaptive one [13, 62, 65]. For the assessment of IC, preintervention and postintervention, we used the following:

• Continuous attention test (CAT): The test, inspired by the second version of the Conners continuous performance test, requires users to press on the screen as fast as possible when a letter appears, unless the letter is “X.” Letters appear at different time intervals (1, 2, and 3 s) for 15 min. After the assessment, the test provides different continuous attention‐derived measures, such as omissions (the student fails to react to a stimulus/letter), commissions (the student reacts falsely to the letter X while they should just wait for it to disappear), and reaction time. A postintervention reduction in omissions and commissions suggests that the intervention is successful in training (and improving) executive functions, especially IC as well as attention.
• Cognitive assessment battery by Cognifit: The battery assesses inhibition control with three activities, including Stroop test.

#### 2.4.3. Mental Flexibility

The term refers to the ability of a person to shift between mental states, rule sets, and tasks [54], as well as transition from one thought to another and the use of flexible problem‐solving ability in a variety of contexts [59]. For the assessment, we used the following:

• Trail making test A and B: These tests are used as screening tools that measure processing speed, sequencing, mental flexibility, and visual‐motor skills [39, 60]. The test is given in two parts. For Part A, students are required to connect numbered circles presenting numbers ranging from 1 to 25. For Part B, students are required to connect circles presenting numbers and letters interchangeably—from a total of 24 circles, half of them presenting numbers ranging from 1 to 12 and the other half presenting letters from A to M (Greek alphabet). The test is easy to implement and takes less than 5 min. Students are timed for both parts. The test is given as a baseline measurement for mental flexibility and postintervention as a measure of potential changes in EF.
• Cognitive assessment battery by Cognifit: The battery assesses mental flexibility under the name shifting and within the broader concept of reasoning. It provides three activities for the assessment. It is used pre and postintervention and potential changes in the students′ cognitive profile are evaluated. Better postintervention performance indicates a positive effect of the Intervention.

### 2.5. Statistical Analysis

The statistical analysis was conducted using IBM SPSS Statistics 25.0. A significance level of *p* < 0.05 was used. Continuous variables used in the study are described using measures of central tendency (mean, median) and measures of dispersion (interquartile range, standard deviation, minimum, and maximum values), whereas qualitative variables are expressed as counts and percentages of participants in each category.

Subsequently, the normality assumption for the quantitative variables was tested, as the choice of appropriate statistical tests is determined based on the validity of this assumption. The normality assumption for the variables was tested using the Shapiro–Wilk test for samples under 30 units and the Kolmogorov–Smirnov test for samples over 30. The nonparametric Wilcoxon signed‐rank test was used to test the equality of distribution of continuous or ordinal variables in two dependent samples, as it does not assume data normality. Additionally, the Pearson chi‐square test was used to evaluate potential differences between two qualitative variables.

## 3. Results

### 3.1. Participants

An initial interest in participating was expressed by (the parents of) 92 students. Of them, seven were rejected as they did not fulfil inclusion criteria—all of them were students of Special Education with a diagnosis of mild to severe cognitive impairments. Nine of the potential participants did not respond to communication efforts after their initial interest. The remaining 69 students were enrolled in the first phase of the intervention, started with an initial interview and concluded with students and carers signed consent. At this stage, seven participants withdrew their interest and the remaining 74 were enrolled in the phase of preintervention assessment. Another 15 participants dropped out of the study during the intervention or were not included in the results analysis because they completed less than 10 on‐line cognitive training sessions. All students included in the sample completed academic skills training. The total sample size was 54 students.

#### 3.1.1. Participants′ Characteristics

Sample consists of 54 students with the majority of male students (66.7%, *N* = 36). Mean age of participants was 14.38 years. ADHD—inattentive type was the first diagnosis for 36.4% of students compared with 34.5% ADHD—hyperactive/impulsive type. Of the 54 students, 32% (*N* = 17) had a second diagnosis of another neurodevelopmental disorder.

Most of participant students came from two‐parent family (both parents living together), 74.5% (*N* = 41), whereas their family income was above average, 43.2% (*N* = 21) as compared with the Cyprus average personal income, which is calculated to the amount of €24 300 (gross). The age of parents was 40–49 years for 81.1% (*N* = 43) of mothers and for 75% (*N* = 36) of fathers. The lowest completed level of education was a university degree for 44.9% of mothers and for 44.7% of fathers (*N* = 22 and 21, respectively). None of the parents was unemployed at the time of the research.

Regarding the presence of family history of ADHD or other conditions, none of the parents were reported to have a diagnosis of ADHD, whereas only one father was reported to have a diagnosis of neurodevelopmental disorder—specifically a specific learning disorder. Nevertheless, there was suspected ADHD for 28% (*N* = 14) of mothers and 25.5% (*N* = 12) of the fathers. Confirmed ADHD diagnosis was given to one or more of the siblings of 13.2% of students—participants (*N* = 7) and a suspected diagnosis for the sibling(s) of the 22.6% of the student‐participants.

At the time of the students′ express of interest to participate, their history regarding treatments and/or training revealed a majority of adolescent students who never received any kind of treatment/intervention/training, neither at their childhood nor at their current age. This is displayed on Table 2, and it reveals a tendency to reduce nonpharmacological treatments and to increase medication as a sole treatment, though the percentage of students taking medication remains low.

**Table 2:** Treatment history.

Intervention	During childhood (< 13 years) % (*N*)	Currently (13+) % (*N*)
Only medication	1.8 (1)	9.1 (5)
Medication PLUS other interventions	5.5 (3)	3.6 (2)
Combination of interventions without medication	18.2 (10)	7.2 (4)
None	72.7 (40)	76.4 (42)
Other/nonspecified/missing information	1.8 (1)	3.6 (2)

### 3.2. Effects on Executive Functions and Cognitive Abilities

A Wilcoxon signed‐rank test was conducted to evaluate the impact of the intervention on a range of cognitive measures. Statistically significant improvements were observed in several domains. Participants demonstrated gains in short‐term memory as measured by digit span forward (*z* = –1.983, *p* = 0.047) and WM via digit span backwards (*z* = –1.977, *p* = 0.048). Mental flexibility showed a notable increase on the digit span with distractions (*z* = –2.457, *p* = 0.014) and Trail Making B (*z* = –4.053, *p* < 0.001), while processing speed improved significantly on Trail Making A (*z* = –3.746, *p* < 0.001). WM enhancement was further supported by results from the CAB WM task (*z* = –3.224, *p* = 0.001). Reductions in omission errors on the CAT (*z* = –1.992, *p* = 0.046) also indicated better sustained attention.

Although not statistically significant, numerical trends were observed in CAT Commissions (*p* = 0.068), CAT reaction time (*p* = 0.074), CAB IC (p = 0.089), and CAB Short‐term memory (*p* = 0.131), suggesting potential areas for further exploration. Measures of planning and focused attention did not change meaningfully across time points.

Table 3 below presents performance on the digit span test across three conditions, before and after the intervention, providing a more detailed examination of changes in WM.

**Table 3:** Performance on the digit span test (three variations), before and after the intervention.

	Preintervnetion *N* = 51	Postintervention *N* = 27			
	Median (Q1–Q3)	Median (Q1–Q3)	*z*	*p*	*r*
Digit span forward	4 (3–4)	4 (4–4)	−1.983	**0.047**	0.38
Digit span backwards	3 (2–4)	4 (2–4)	−1.977	**0.048**	0.38
Digit span distraction	2 (1–3.3)	3 (2–4)	−2.457	**0.014**	0.47

*Note:* Bold *p* values indicate statistically significant differences (*p* <0.05). Effect sizes (r) represent the magnitude of change between preintervention and postintervention. Significant results (*p* =0.047, *p* =0.048 and *p* =0.014) indicate improvement from preintervention to postintervention in working memory domains. Effect sizes (*r* =0.38‐0.47) indicate moderate practical significance across all significant outcomes.

Median scores and interquartile ranges are presented alongside Wilcoxon signed‐rank test statistics (*z*), significance values (*p*), and effect sizes (*r*). Digit span forward presents a statistically significant (*p* = 0.407) change in preintervention and postintervention scores, with a moderate effect size (*r* = 0.38). This reflects a meaningful enhancement in WM.

Digit span backwards also presents a statistically significant (*p* = 0.048) change in preintervention and postintervention scores, with a moderate effect size (*r* = 0.38). This reflects a meaningful enhancement in WM.

Finally, digit span with distraction presents a statistically significant (*p* = 0.014) change in preintervention and postintervention scores, with a moderate‐to‐large effect size (*r* = 0.47). This reflects a substantial improvement in divided attention and inhibition control.

Following, Table 4 summarizes performance on the Trail Making Test—Parts A and B, before and after the intervention. Results summarized on Table 4 highlight changes in processing speed and cognitive flexibility.

**Table 4:** Performance on the Trail Making Test Part A and Part B, before and after the intervention.

	Preintervention	Postintervention			
	Median (Q1–Q3)	Median (Q1–Q3)	*z*	*p*	*r*
Trail Making A (sec)	31 (23–40)	23 (19–31.8)	−3.746	**< 0.001**	= 0.80
Trail Making B (sec)	82 (64–102)	61.5 (56.3–77.3)	−4.053	**< 0.001**	= 0.86

*Note:* Bold *p* values indicate statistically significant differences (*p* <0.05). Effect sizes (r) represent the magnitude of change, with values ≥0.50 indicating large effects. Both trail making conditions showed statistically significant improvements with large effect sizes, indicating substantial gains in processing speed and cognitive flexibility.

Median scores and interquartile ranges are presented alongside Wilcoxon signed‐rank test statistics (*z*), significance values (*p*), and effect sizes (*r*). Performance scores on the Trail Making Test, both Part A and Part B, showed statistically significant improvements following the intervention (for both *p* < 0.001), reflecting a decrease in time of completion. The results indicate enhanced processing speed and cognitive flexibility. Both effect sizes for Part A and Part B were large (*r* = 0.80 and *r* = 0.86, respectively), suggesting that the intervention produces robust and clinically meaningful gains in EF and specifically on cognitive flexibility.

Performance on the CAT before and after the intervention is presented on Table 5. Performance on CAT illustrates changes in sustained attention, IC, and reaction time across assessment points.

**Table 5:** Performance scores on the continuous attention test (CAT) before and after the intervention.

	Preintervention	Postintervention			
	Median (Q1–Q3)	Median (Q1–Q3)	*z*	*p*	*r*
CAT omissions	2.5 (1.5–4.7)	1 (0.3–3.4)	−1.992	**0.046**	0.38
CAT commissions	66 (47.2–87.1)	59.7 (33.2–81.2)	−1.822	0.068	0.34
CAT reaction time	467 (405–504.5)	472 (442.8–520.3)	−1.784	0.074	0.34

*Note:* Bold *p* values indicate statistically significant differences (*p* <0.05); nonbold values indicate nonsignificant results. Effect sizes (r) indicate magnitude of change (≈0.10 small, ≈0.30 moderate, ≥0.50 large). A statistically significant reduction was observed only for CAT omissions, with a moderate effect size, whereas commissions and reaction time did not show significant changes.

Median scores and interquartile ranges are reported alongside Wilcoxon signed‐rank test statistics (*z*), significance values (*p*), and effect sizes (*r*). A statistically significant reduction was observed in omission errors (*p* = 0.046, *r* = 0.38), indicating improved sustained attention. Commissions and reaction time showed nonsignificant trends toward improvement (*p* = 0.068 and *p* = 0.074, respectively), each with moderate effect sizes (*r* = 0.34), suggesting partial but inconsistent gains in IC and processing speed.

Table 6 summarizes preintervention and postintervention performance scores across five subtests of the CAB, measuring performance on four cognitive domains: planning, WM, short‐term memory and focused attention.

**Table 6:** Cognitive performance on CAB subtests before and after the intervention.

	Preintervention	Postintervention			
	Median (Q1–Q3)	Median (Q1–Q3)	*z*	*p*	*r*
Planning	540 (240.5–700)	604 (404.5–711.5)	−1.224	0.221	0.29
Working Memory	307 (169.5–483)	388 (300–570)	−3.224	**0.001**	0.76
Short‐term Memory	407 (192–590.5)	476 (248.5–647)	−1.510	0.131	0.36
Focused Attention	458 (258–610)	476 (324–606.5)	−0.188	0.851	0.04

*Note:* Bold *p* values indicate statistically significant differences (*p* <0.05); nonbold values indicate nonsignificant results. Effect sizes (r) indicate magnitude of change (≈0.10 small, ≈0.30 moderate, ≥0.50 large). Working memory demonstrated a statistically significant improvement with a large effect size, whereas planning, short‐term memory, and focused attention did not show significant changes.

Median scores and interquartile ranges are presented alongside Wilcoxon signed‐rank test statistics (*z*), significance values (*p*), and effect sizes (*r*). Scores in WM show a statistically significant improvement (*p* = 0.001, *r* = 0.76) indicating large gains in storing and manipulating information. A trend toward improvement, though nonsignificant and with moderate effect sizes, can be observed for short‐term memory (*p* = 0.131, *r* = 0.36) and planning skills (*p* = 0.221, *r* = 0.29), suggesting that the intervention did not meaningfully affect those skills.

In regard to the stated hypotheses of the current research, we observe that Hypothesis 1 is accepted as WM did show statistically significant improved scores, as measured preintervention and postintervention by both digit span backwards and Cognifit Assessment Battery. Hypothesis 2 is also accepted as Trail Making B scores showed statistically significant improvement, revealing an improvement in mental flexibility.

Hypothesis 3 stated that IC would be significantly improved because of the MOTI. In fact, two assessment tools give statistically significant improved scores on IC, and two more give near‐significance results, showing an improvement. Thus Hypothesis 3 is partly accepted.

Hypothesis 4 is partly accepted since Trail Making part A and CAT omissions gave statistically significant results for the pre–postintervention scores of processing speed. On the other hand, CAT reaction time gave a result indicative of improvement on reaction time, but this is not statistically significant.

## 4. Discussion

The MOTI under investigation combines three intervention methods: (a) cognitive training—which is considered to be in the heart of the protocol, (b) academic skills training, which is complementary to cognitive training in the sense that “translates” the cognitive training (potential) gains into real‐life, and (c) parents′ psychoeducation, which is complementary to the other two interventions and not mandatory.

Cognitive training is at the heart of intervention because of the critical importance of cognitive skills in all aspects of human functioning. Executive functions play a foundational role in everyday functioning and participation by enabling individuals to sustain attention, follow instructions, apply knowledge, think creatively, and adhere to social expectations [10]. These cognitive abilities are particularly essential for successful engagement in academic settings, which represent a primary occupation during childhood and adolescence.

Several studies (e.g., [12, 62–65 ]) suggest that there is a need to work on EF (and other cognitive structures) as it is a crucial aspect of the management of ADHD. Cognitive training is effective in reducing symptoms of ADHD and in improving functioning as it targets deficits characteristic of the condition, such as attentional control, WM, and IC [66]. A well‐developed state of EF permits individuals to manage age‐related tasks with an appropriate degree of independence [67].

As mentioned by Gunzenhauser and Nuckles [33], the ultimate purpose of EF training is the improvement of real‐life goal attainment. On the same route, MOTI is aimed at enhancing the students′ ability to thrive in challenging yet important settings like school. Improving WM, mental flexibility, IC, processing speed and reaction time we expect to have a direct impact on crucial indicators of successful school participation, including on‐task behaviors, following rules of the academic setting, attention, response time.

Our results show a significant improvement in several areas of cognitive functioning, including WM, mental flexibility, and IC, along with processing speed and reaction time. These results are in accordance with previous research reporting positive effects on adolescents.

Academic skills training complements cognitive training by strengthening the transfer of acquired skills to real‐world settings and daily occupational demands. As emphasized by Gunzenhauser and Nuckles and Blair [32, 33], successful far‐transfer of executive function (EF) training depends on practicing cognitive strategies within authentic, meaningful contexts where they are intended to be used. Moreover, Rios‐Davis et al. [68] recommend a hybrid intervention model for adolescents—one that merges operant techniques common in early childhood interventions with the explicit teaching of coping strategies typically employed with adults [22]. The academic skills training component of the MOTI program embodies this approach: following cognitive training in a clinical environment, students receive direct, structured guidance on applying and extending these skills within academic settings.

Our results show that cognitive training interventions, when complemented by training the targeted skills in real‐life situations, yield important positive effects for adolescents with ADHD. This is an important contribution to existing knowledge, as existing research within the field of OT is limited. A research review reveals that studies in OT for the age range of 12–18 years are scarce and any evidence supporting cognitive interventions is mostly derived from studies with younger children.

Our results show a significant improvement on test scores, as compared preintervention and postintervention. This allows for the assumption that the intervention protocol was successful in training WM for adolescent students with ADHD. In the same line, significant pre‐to‐post improvements in EF, as measured by the Behavior Rating Inventory of Executive Function, which includes a WM subscale, are presented by the study of Levanon‐Erez et al. regarding the effectiveness of the Cognitive‐Fucntional Program (Cog‐Fun) [7]. In their feasibility study of 22 adolescents (13–18 years), Levanon‐Erez et al. reported medium‐to‐large effect sizes on overall EF and strategy use, suggesting transfer into daily tasks that rely on WM [7] *Pomodoro*. Overall, despite the fact that OT interventions very often have enhancing WM within their priorities, research in the field is limited on children and adolescents as young as 14 years old, and despite our efforts, it was not possible to spot any other study utilizing cognitive training for the age range and diagnosis as our research. In that sense, our findings contribute in a unique way to existing knowledge.

## 5. Conclusions

The preliminary results largely support the research hypothesis, demonstrating statistically significant improvements across trained executive function components and untrained cognitive abilities.

The findings confirm that the multicomponent intervention protocol (MOTI) effectively strengthens core executive functions, aligning with the initial hypothesis. The statistical significance of these results suggests that targeted cognitive training and structured interventions can lead to measurable gains in EF‐related skills among adolescents with ADHD.

The adoption of the multicomponent intervention protocol in OT practice can help provide comprehensive treatment for adolescents with ADHD.

### 5.1. Limitations and Future Directions

This study presents several limitations. One major constraint was the inability to collect sufficient postintervention data to conduct meaningful analyses on outcomes such as quality of life and academic achievement, particularly in relation to the potential impact of the MOTI on adolescents with ADHD. Closely related is a second limitation—the lack of conclusive findings regarding far‐transfer effects and the translational applicability of the MOTI. These gaps highlight important directions for future research.

Another significant limitation was the absence of long‐term follow‐up data. Although a reassessment was initially planned for 3 months postintervention, logistical challenges emerged as many adolescents relocated for studies or faced demanding academic schedules, leading to reluctance in participating in follow‐up assessments. This issue reflects a broader concern of treatment nonadherence, which warrants further exploration.

Additionally, the small sample size may limit the generalizability of the findings and contributed to difficulties in securing participants for long‐term evaluation. Closely related, it is acknowledged that the lack of a control group in combination with the single‐group pretest/posttest design might limit causal inference. Though some threats to internal validity are likely minimal in our study, for example, maturation effect as spontaneous improvement in EF is not typically expected within the timeframe of +/−8 weeks that intercedes between the preintervention and postintervention assessment, alternative explanations cannot be fully excluded, and the results should be interpreted with the appropriate caution. Future research should incorporate a control group either as a waiting list or as treatment‐as‐usual to facilitate better isolation of the effects of the intervention. Conducting supplementary analyses to explore whether greater exposure to intervention is associated with stronger gains, would be useful strategy.

As a preliminary investigation, this study offers a foundational framework and indicative insights. The results should be viewed as exploratory, with the understanding that future studies employing more rigorous methodologies may yield deeper and more definitive conclusions. One of the possible future studies might investigate how specific components of the MOTI might be more strongly associated with specific EF gains or with specific improvements in functionality.

In a future study, the addition of a control group either in the form of waiting list or treatment‐as‐usual and/or conduct of sensitivity analyses investigating duration of intervention and its relation to potential changes.

## Author Contributions

**Elisavet Pavlou Papagianni:** conceptualization, investigation, methodology, writing—original draft, writing—review and editing, and project administration. **Eleni Theodorou:** supervision, methodology, and writing—review and editing.

## Funding

No funding was received for this manuscript.

## Ethics Statement

The was approved by the Cyprus National Bioethics Committee—reg. Number: CNBC/EP/2021/10 (11/06/2021) and the Centre for Academic Research and Evaluation of the Cyprus of Education, Sports and Youth—reg. Number: 7.15.01.25.8.2/6 (11/08/2021).

## Consent

Informed written consent was obtained by all participants and their parents (or guardians).

## Conflicts of Interest

The authors declare no conflicts of interest.

## Data Availability

The datasets analyzed in this study are not publicly available due to data protection requirements. However, anonymized data or files may be available from the corresponding authors upon reasonable request.
